# Association between blood mitochondrial DNA copy number and cognitive function: A cross-sectional study based on the Yakumo Study

**DOI:** 10.20407/fmj.2025-038

**Published:** 2026-02-28

**Authors:** Atsushi Teshigawara, Genki Mizuno, Hiroya Yamada, Yoshiki Tsuboi, Eiji Munetsuna, Yuji Hattori, Mirai Yamazaki, Yoshitaka Ando, Itsuki Kageyama, Takuya Wakasugi, Hayato Ichikawa, Hiroshi Okumiyama, Ryosuke Fujii, Akihiko Iwahara, Takeshi Hatta, Hiroaki Ishikawa, Koji Ohashi, Koji Suzuki

**Affiliations:** 1 Department of Preventive Medical Sciences, Fujita Health University, School of Medical Sciences, Toyoake, Aichi, Japan; 2 Department of Medical Technology, Tokyo University of Technology School of Health Sciences, Ota, Tokyo, Japan; 3 Department of Hygiene, Fujita Health University, School of Medicine, Toyoake, Aichi, Japan; 4 Department of Animal Science and Biotechnology, Azabu University School of Veterinary Medicine, Sagamihara, Kanagawa, Japan; 5 International Center for Cell and Gene Therapy, Fujita Health University, Toyoake, Aichi, Japan; 6 Department of Informative Clinical Medicine, Fujita Health University, School of Medical Sciences, Toyoake, Aichi, Japan; 7 Department of Public Health, Aichi Medical University School of Medicine, Nagakute, Aichi, Japan; 8 Department of Psychology and Collaboration, Kyoto Women’s University Faculty of Psychology and Collaboration, Kyoto, Kyoto, Japan; 9 Department of Health Sciences, Kansai University of Welfare Sciences Faculty of Health and Welfare, Kashiwara, Osaka, Japan

**Keywords:** Mitochondria, Mitochondrial DNA copy number, Cognitive function, Peripheral blood, Biomarker

## Abstract

**Objectives:**

Mitochondrial dysfunction has been implicated in neurodegenerative diseases, but evidence regarding its association with cognitive performance in the general population remains limited. This study aimed to examine the association between peripheral blood mitochondrial DNA copy number (mtDNA-CN) and cognitive function in the general Japanese population.

**Methods:**

We conducted a cross-sectional analysis of 282 participants (134 men and 148 women) from the Yakumo Study, a population-based health examination in Hokkaido, Japan. Peripheral blood mtDNA-CN was measured by quantitative real-time PCR and categorized into tertiles. Cognitive function was assessed using the short version of the Mini-Mental State Examination (SMMSE), the Logical Memory Test (LMT), and the Digit Cancellation Test (D-CAT). Logistic regression analyses were performed to evaluate the association between mtDNA-CN levels and cognitive performance, with adjustments for relevant demographic and clinical factors.

**Results:**

Lower mtDNA-CN was significantly associated with poorer SMMSE scores in women and with reduced D-CAT3 performance—reflecting attention and executive function—in men. No significant associations were observed for LMT scores in either sex. These domain- and sex-specific associations remained consistent after adjustment for potential confounders.

**Conclusions:**

Lower mtDNA-CN was associated with poorer cognitive performance in the general Japanese population, in a cognitive domain- and sex-specific manner. mtDNA-CN thus has potential as a non-invasive biomarker for the early identification of individuals at increased risk of cognitive decline. Longitudinal studies are necessary to evaluate its predictive utility and potential application in dementia prevention strategies.

## Introduction

The global prevalence of dementia is rapidly increasing in parallel with the acceleration of population aging. According to the World Health Organization (WHO), approximately 55 million people are currently living with dementia worldwide, and nearly 10 million new cases are diagnosed annually.^1^ The rise in dementia cases poses not only a serious threat to individual quality of life but also a substantial burden on families, communities, and health and long-term care systems.^2^ In Japan, where older adults aged 65 years and above account for approximately 30% of the total population, the prevalence of dementia is expected to continue increasing in the coming decades.^3^ In this context, there has been growing interest in mild cognitive impairment (MCI), a transitional state between normal aging and dementia.^4^ Although individuals with MCI maintain independence in daily life, they exhibit objectively measurable cognitive decline.^5,6^ Timely identification of individuals at risk during this stage is considered critical for preventing or delaying progression to dementia.^7^ Therefore, the establishment of simple, objective, and cost-effective biomarkers for the early detection of cognitive decline is a high public health priority.

Mitochondria, which play a key role in energy metabolism, oxidative stress regulation, and apoptosis, are essential for the maintenance of neuronal function, especially given the high energy demands of neurons.^8–10^ Mitochondrial dysfunction has been implicated in age-related cognitive decline and neurodegenerative diseases.^11^ Mitochondrial DNA copy number (mtDNA-CN), measured in peripheral blood mononuclear cells, is considered a surrogate marker of systemic mitochondrial function.^8,12^ A reduction in mtDNA-CN may impair synaptic plasticity and neurotransmission, potentially leading to cognitive impairment.

Various studies conducted in Western populations have reported associations between reduced levels of mtDNA-CN in blood and the development of MCI or Alzheimer’s disease.^13–16^ However, most studies of this kind were conducted in Western populations, such as those included in the Health and Retirement, and often focused on older adults or patients with cognitive impairment.^13–15^ As such, these associations are still not well understood in Asian populations,^16^ including among the Japanese. Considering the potential influence of ethnic differences, lifestyle factors, and genetic background including mitochondrial DNA polymorphisms on cognition, there is a need for further studies in diverse populations in order to obtain a comprehensive understanding of how cognitive impairment develops.

Most previous studies relied solely on the Mini-Mental State Examination (MMSE) to measure cognition, which primarily captures orientation and verbal fluency, and may lack sensitivity to other important cognitive domains.^17^ As such, domains such as episodic memory, attention, and processing speed are underrepresented in the literature on studies measuring cognitive decline.

We therefore conducted a cross-sectional study in a sample of Japanese adults to investigate the association between blood mtDNA-CN and multiple cognitive function measures. Specifically, we used the MMSE for global cognitive screening, the Logical Memory Test (LMT) to assess episodic memory, and the Digit Cancellation Test (D-CAT) to evaluate attention and processing speed.^18–20^ By incorporating a multidimensional assessment of cognitive domains, this study is intended to provide a comprehensive analysis of the relationship between mtDNA-CN and cognitive function in the general population.

## Methods

### Study participants

This cross-sectional study was conducted in Yakumo, a town in Hokkaido, Japan, using data obtained from annual health examinations. The Yakumo Study, an ongoing community-based cohort initiated in 1982, has served as a platform for numerous epidemiological investigations.^21–23^ In 2015, a total of 525 residents participated in the health checkups; of these, 282 individuals who completed the cognitive assessments and provided informed consent were included in the present analysis. Public health nurses measured height, weight, and blood pressure, and collected fasting blood samples as well as self-administered questionnaires regarding participants’ demographics, lifestyle factors, and medical history. Questionnaire items included age, sex, smoking status (never, ever, or current), and educational attainment (<9 years, 9–12 years, or >12 years of education). This study was approved by the Ethics Committee of Fujita Health University, and written informed consent was obtained from all participants.

### Measurement of blood biochemical parameters

Fasting venous blood samples were collected and centrifuged within 1 h of collection. Serum was aliquoted and stored at –80°C until analysis. Routine biochemical analyses and complete blood counts were performed using an automated analyzer at the Yakumo General Hospital Laboratory.

### DNA extraction from peripheral leukocytes and measurement of mitochondrial DNA copy number

Blood samples were drawn into disodium EDTA tubes and then centrifuged at 1500 g for 10 min. Genomic DNA was then extracted from peripheral leukocytes using NucleoSpin Tissue kits (Takara, Kusatsu Japan).^24^ After extraction, the DNA concentration was measured using a NanoDrop ND-1000 spectrophotometer (NanoDrop Technologies, Wilmington, DE, USA). Tris-EDTA buffer was used to adjust the concentration to 100 ng/μL, followed by storage at –80°C until measurement. The mtDNA-CN was measured as described above by real-time polymerase chain reaction (PCR).^25^ The primers used for the mtDNA-CN assay were as follows: mitochondrial-NADH dehydrogenase subunit 1 (mt-ND1), 5'-CACCCAAGAACAGGGTTTGT-3' (forward), and 5'-TGGCCATGGGTATGTTGTTAA-3' (reverse); Actin beta (Actb), 5'-ACCCACACTGTGCCCATCTAC-3' (forward) and 5'-TCGGTGAGGATCTTCATGAGGTA-3' (reverse).^25^ The mtDNA-CN was calculated from the ratio of mt-ND1, the mitochondrial genomic DNA, to Actb, the nuclear genomic DNA.

### Cognitive function tests

We administered three cognitive assessments to those undergoing the voluntary health checkups: the short version of the Mini-Mental State Examination (SMMSE), the LMT, and the D-CAT. Each cognitive function test is described below.

### Short version of the Mini-Mental State Examination (SMMSE)

The SMMSE was developed by Nagoya University Cognitive Evaluation Battery and is a simplified version of the original MMSE that excludes questions that are assumed to be answerable by subjects who can undergo medical checkups on their own. In this analysis, as in previous studies, the SMMSE cut-off value for determining early cognitive decline was set at 27/28, and subjects scoring 27 or less were defined as the early cognitively impaired group.^26^

### Logical Memory Test (LMT)

For the Logical Memory Test, examinees were assessed using the Japanese version of the Wechsler Memory Scale-Revised (WMS-R). In this assessment, the examiner read a passage of narrative prose twice and asked the subject to repeat phrases from it immediately afterward. Although two types of repetition of this kind in typical logical memory tests, immediate and delayed playback, only immediate playback was performed in this study.^18^ The scoring criterion was based on the number of phrases that could be playbacked by dividing the prose into 25 phrases, which were scored on a 25-point scale: A lower score was considered to represent poorer memory. The analysis was conducted by classifying those with the lower 25% of test scores into the “suspected cognitive decline group” and the rest as the “normal group.”

### Digit Cancellation Test (D-CAT)

D-CAT assesses three levels of attention, namely, focal, sustained, and selective attention, as representative of prefrontal function.^18–20^ In D-CAT, the subject is presented with a random sequence of numbers. The subject is then given a target digit and must cross out all instances of it from the list of numbers as quickly as possible within 1 min. In the Yakumo Study, the one-letter peripheral test (D-CAT1; with one Arabic digit as the target digit) was administered, followed by the three-letter peripheral test (D-CAT3; with three Arabic digits as the target digits). Regarding the difference between these peripheral tests, the one-letter peripheral test is thought to measure focal and sustained attention and processing speed. Meanwhile, D-CAT3, in addition to D-CAT1, is related to memory retention and the need to complete the assessment while remembering three numbers. As above, the analysis was conducted by classifying those with the lower 25% of scores into the “suspected cognitive decline group” and the rest as the “normal group.”

### Statistical analyses

Statistical analyses were conducted using JMP version 14.2.0 (SAS Institute, Cary, NC, USA). Continuous variables were expressed as mean±standard deviation or as geometric mean with interquartile range and were compared using Student’s t-test or Wilcoxon’s rank-sum test, as appropriate. Categorical variables were compared using Pearson’s chi-square test. Participants were categorized into three groups based on the distribution of blood mtDNA-CN levels: low (lowest 25%), middle (25th–75th percentile), and high (highest 25%). To examine the associations between mtDNA-CN and cognitive function, binary logistic regression analyses were performed to estimate odds ratios (ORs) and 95% confidence intervals (CIs) for suspected cognitive impairment, with the high mtDNA-CN group used as the reference category. Three models were constructed: Model 1, unadjusted; Model 2, adjusted for age and educational attainment (<9 years, 9–12 years, and >12 years of education); and Model 3, further adjusted for smoking status (never, ever, and current), systolic blood pressure, low-density-lipoprotein cholesterol, and platelet count. All statistical tests were two-tailed, and a *P*-value <0.05 was considered statistically significant.

## Results

Significant sex differences were observed in various biochemical parameters, lifestyle factors such as smoking and alcohol consumption, and cognitive function scores (Table 1). Among these, blood mtDNA-CN levels were significantly higher in women than in men. Given these differences, all subsequent analyses were stratified by sex.

Participants were categorized into three groups based on blood mtDNA-CN levels: low (lowest 25%), middle (25th–75th percentile), and high (highest 25%). Table 2 presents the characteristics of male participants stratified by mtDNA-CN levels. Significant group differences were observed in LMT, D-CAT1, and D-CAT3 (*P*=0.005, *P*<0.001, and *P*=0.001, respectively). No significant differences were found in any other background or clinical parameters. Table 3 shows the corresponding data for female participants. Apart from mtDNA-CN itself, no significant differences were observed in any variables, including cognitive test scores.

Table 4 shows the results of logistic regression analyses examining the association between mtDNA-CN and cognitive performance among men. In the unadjusted Model 1, individuals in the low mtDNA-CN group had a higher prevalence of low performance on D-CAT1 (39.4%) than those in the middle (20.6%) and high (18.2%) groups (*P* for trend <0.05). A similar trend was observed for D-CAT3 (low: 39.4%, middle: 22.1%, and high: 15.2%, respectively; *P* for trend <0.05). The low mtDNA-CN group showed a significantly increased odds of performing poorly on D-CAT3 compared with the high group (OR [95% CI]: 3.64 [1.12–11.85]). These associations remained robust in the fully adjusted Model 3 (OR [95% CI]: 3.77 [1.01–14.07]). No significant associations were observed for SMMSE or LMT scores. Table 5 shows the results of logistic regression analysis for women, as well as for men. In the unadjusted Model 1, there was no significant difference in the association between mtDNA-CN and SMMSE. In the adjusted Model 2, the low and middle mtDNA-CN groups showed a significantly increased odds of performing poorly on SMMSE compared with the high group (ORs [95% CI]; low group: 5.46 [1.33–22.43], middle group: 4.90 [1.32–18.20]). Similar results were also obtained with Model 3 (ORs [95% CI]; low group: 5.46 [1.28–23.38], middle group: 4.91 [1.29–18.72]). In D-CAT3, the unadjusted Model 1 showed that the low mtDNA-CN group (40.5%) had a higher proportion of attention function decline compared with the middle (20.3%) and high (18.9%) groups (*P* value for trend <0.05). A significant association was observed between the low mtDNA-CN group and cognitive decline (OR [95% CI]; 2.92 [1.02–8.37]). but no significant association was observed in Models 2 and 3.

## Discussion

This study provides evidence that lower mtDNA-CN in peripheral blood is associated with poorer cognitive function in the general Japanese population. The observed associations across multiple cognitive domains suggest that mtDNA-CN may reflect early functional changes relevant to cognitive health. Given its non-invasive and quantifiable nature, mtDNA-CN could serve as a useful biomarker for identifying individuals who may be at increased risk of cognitive decline.^27^ Nevertheless, because this work involved a cross-sectional analysis, longitudinal studies are necessary to determine whether mtDNA-CN has value for predicting future cognitive outcomes.

Previous studies, primarily conducted in Western populations, reported associations between reduced blood mtDNA-CN and cognitive impairment, including mild cognitive impairment and Alzheimer’s disease.^14,15^ For example, Zhang et al demonstrated that lower mtDNA-CN levels were associated with reduced hippocampal volume and poorer cognitive performance.^13^ Similarly, data from the Baltimore Longitudinal Study of Aging indicated that individuals with lower mtDNA-CN had an increased risk of developing Alzheimer’s disease.^28^ However, many of these studies included participants from older age groups, often with dementia or receiving institutional care, which limits their generalizability to the general population.^13–15^ Moreover, the majority of these cohorts comprised individuals of European ancestry, with relatively few studies conducted in Asian populations.^16^ Given the potential influence of ethnicity, genetic background, and lifestyle factors on mitochondrial function—including mtDNA polymorphisms—further research in diverse populations is needed.

The present study extends previous findings by examining the association between mtDNA-CN and cognitive performance in the general Japanese population. Our findings support previous reports showing that lower mtDNA-CN is associated with poorer global cognitive performance,^13–15^ but we also provide additional insight by evaluating multiple cognitive domains. Most previous research relied solely on the MMSE, a general cognitive screening tool.^16,29^ In contrast, we incorporated LMT to assess episodic memory and D-CAT to measure attention and processing speed. Notably, the observed association between lower mtDNA-CN and D-CAT3 performance in men highlights a potential link between mitochondrial dysfunction and executive functioning, a cognitive domain that has received relatively limited attention in the literature. These results suggest that mitochondrial biomarkers may provide insights into specific cognitive processes rather than global decline alone.

Our results also align with previous evidence indicating sex differences in mtDNA-CN, with women generally exhibiting higher levels than men.^12,25^ This difference has been attributed to estrogen-mediated protection against oxidative stress, age-related mitochondrial decline, and the maternal inheritance of mtDNA.^30^ Estrogen enhances mitochondrial biogenesis and maintains mitochondrial integrity, which may contribute to the relative preservation of cognitive function in women.^15,31^ Although estrogen secretion in women gradually declines toward menopause and reaches levels comparable to those in men after menopause, women nevertheless exhibit greater protective effects against oxidative stress than men, independent of estrogen.^32^ It has been suggested that this protective mechanism may play a crucial role in maintaining mitochondrial protective effects. Reiling et al. reported that mtDNA-CN declines with age primarily in men,^33^ while Vyas et al. found consistently higher mtDNA-CN levels in women across ethnic groups.^34^ These findings highlight the importance of conducting sex-stratified analyses when examining mitochondrial biomarkers in cognitive aging.

The sex-specific associations observed in this study may reflect underlying differences in age-related changes across cognitive domains and brain structures.^35^ For example, the MMSE assesses global cognitive functions such as memory, language, and orientation, and previous studies have suggested that women may experience earlier age-related atrophy in the hippocampus and temporal lobes compared with men.^36^ These structural changes may underlie the observed association between MMSE scores and mtDNA-CN in women. In addition, the MMSE exhibits a ceiling effect in cognitively healthy populations, whereby scores cluster near the upper limit, reducing the ability to detect subtle cognitive decline.^37^ The fact that a significant association was still observed in women, despite this limitation, may indicate the presence of early neurobiological vulnerability.

In contrast, the D-CAT evaluates attention and executive function and relies heavily on the activity and integrity of the prefrontal cortex.^20^ This region is particularly vulnerable to age-related decline, and several studies have reported that men may experience earlier and more pronounced deterioration in prefrontal structure and function compared with women.^38,39^ Furthermore, neuroimaging studies have shown that men tend to rely more heavily on prefrontal activation during working memory and attention tasks.^40^ These neurobiological sex differences are consistent with our finding that lower mtDNA-CN was significantly associated with poorer D-CAT3 performance in men only. However, the absence of a significant association in women may reflect insufficient statistical power in stratified analyses, rather than a true lack of association. Taken together, these findings suggest that the relationship between mitochondrial function and cognitive performance may vary by sex and cognitive domain, but further studies with larger sample sizes are needed to confirm these patterns.

Although the underlying biological mechanisms remain to be fully elucidated, mitochondrial dysfunction has been implicated in neurodegeneration through multiple pathways, including impaired ATP production, disrupted calcium homeostasis, oxidative stress, and reduced synaptic plasticity.^8,10,12^ Previous studies have shown that the expression of mitochondrial regulatory genes such as TFAM and PGC-1α is reduced in Alzheimer’s disease, with intermediate levels observed in individuals with MCI.^41^ Moreover, recent work has demonstrated a positive correlation between peripheral blood mtDNA-CN and mitochondrial gene expression in brain tissue, supporting the validity of mtDNA-CN as a peripheral proxy for central mitochondrial health.^37^ Our findings lend support to the hypothesis that systemic mitochondrial dysfunction may contribute to early cognitive decline via impaired bioenergetic capacity and transcriptional regulation.

It is also important to consider that mtDNA-CN is influenced by various lifestyle and environmental factors. Physical activity, dietary habits (e.g., antioxidant intake), sleep quality, and psychological stress have all been shown to affect mitochondrial function.^34^ Additionally, exposure to environmental toxins such as heavy metals, endocrine-disrupting chemicals, and lifestyle factors like smoking and excessive alcohol consumption may impair mitochondrial biogenesis through oxidative damage.^12,16^ These cumulative exposures may contribute to interindividual variation in mtDNA-CN and could have led to its emerging role as a potential biomarker of biological aging. Thus, the observed associations in this study may partially reflect long-term lifestyle and environmental influences on mitochondrial integrity.

From a public health perspective, mtDNA-CN, as a non-invasive and quantifiable biomarker, may hold promise as a useful tool for the early identification of individuals at risk for cognitive decline.^13,42^ Current diagnostic approaches for MCI often rely on neuropsychological testing, which can be time-consuming, resource-intensive, and less accessible in real-world settings. In contrast, this blood-based biomarker could offer a scalable and objective alternative for population-level screening. If validated in longitudinal studies, mtDNA-CN may support the timely identification of cognitively vulnerable individuals at a stage when lifestyle or pharmacological interventions are most likely to prevent or delay the onset of dementia.

This study had several limitations. First, owing to the cross-sectional design of this work, causal relationships could not be established. Second, the sample size was modest, particularly within subgroups, limiting statistical power. Third, since the present study was conducted among residents of a largely rural area, the findings may not be fully representative of the entire Japanese population. Further analyses in various regions of Japan, including urban areas, are warranted. Fourth, mtDNA-CN was measured in peripheral blood, which may not fully represent mitochondrial dynamics within the central nervous system. Nonetheless, previous evidence supports a correlation between peripheral and central mtDNA-related activity.^27^

In conclusion, this study provides evidence that lower mtDNA-CN is associated with poorer cognitive function among Japanese adults, in a sex- and domain-specific manner. Specifically, reduced mtDNA-CN was linked to lower global cognitive performance in women and poorer attention and executive function in men. These findings support the potential utility of mtDNA-CN as a non-invasive biomarker for early cognitive vulnerability and highlight the need for longitudinal studies to evaluate its role in dementia prevention and to further elucidate the underlying biological pathways.

## Figures and Tables

**Table 1:** Characteristics of study participants

	Men	Women	*P*
n	134	148	
Age (years)	64.4±10.0	62.4±10.1	0.107^a^
LDL cholesterol (mg/dL)	124.4±32.3	130.5±31.1	0.106^a^
Platelet counts (×10^4^/μL)	21.7±5.2	21.9±4.8	0.748^a^
SBP (mmHg)	133.8±19.9	125.6±20.1	<0.001^a^
Smoking habit, n (%)			
never	31 (23.1)	114 (77.0)	<0.001^c^
ever	72 (53.8)	22 (14.9)	
current	31 (23.1)	12 (8.1)	
Education history, n (%)			
9 years >	37 (28.4)	33 (22.9)	0.465^c^
9–12 years	57 (43.9)	63 (43.8)	
12 year <	36 (27.7)	48 (33.3)	
Relative level of mtDNA-CN	1.00±0.23	1.09±0.25	0.003^a^
SMMSE score	27.8 (26.0–30.0)	28.7 (28.0–30.0)	0.002^b^
LMT score	12.5±5.1	14.9±5.1	<0.001^a^
D-CAT1 score	257.6±62.9	292.7±71.7	<0.001^a^
D-CAT3 score	159.6±38.3	181.3±43.1	<0.001^a^

Values are mean±SD, geometric mean (25th–75th percentiles), or n (%). Statistically significant. a: student t, b: Wilcoxon, c: Peason chi-square tests. Abbreviations: LDL; low-density lipoprotein, SBP; systolic blood pressure, mtDNA-CN; mitochondrial DNA copy number, SMMSE; Short version of Mini-Mental State Examination, LMT; Logical Memory Test, D-CAT; Digit Cancellation Test.

**Table 2:** Characteristics of study participants in stratified mtDNA-CN in men

	Men			
	mtDNA-CN			
	Low (n=33)	Middle (n=68)	High (n=33)	*P*
Age (years)	65.4±8.2	65.1±10.4	61.8±10.7	0.237^a^
SBP (mmHg)	136.2±18.1	130.3±20.0	138.6±20.8	0.103^a^
Platelet count (×10^4^/μL)	21.7±5.6	21.5±5.4	22.2±4.6	0.845^a^
LDL cholesterol (mg/dL)	121.7±30.8	121.2±32.9	133.5±31.6	0.171^a^
Smoking habit, n (%)				
never	10 (30.3)	13 (19.1)	8 (24.2)	0.577^c^
ever	18 (54.6)	36 (53.0)	18 (54.6)	
current	5 (15.1)	19 (27.9)	7 (21.2)	
Education history, n (%)				
9 years >	10 (31.3)	23 (35.4)	4 (12.1)	0.070^c^
9–12 years	16 (50.0)	22 (33.9)	19 (57.6)	
12 year <	6 (18.7)	20 (30.7)	10 (30.3)	
Relative level of mtDNA-CN	0.55±0.12	0.77±0.09	1.00±0.10	<0.001^a^
SMMSE score	27.5 (25.0–30.0)	27.8 (26.0–30.0)	28.5 (27.0–30.0)	0.073^b^
LMT score	10.1±5.2	13.6±4.9	12.6±4.9	0.005^a^
D-CAT1 score	227.3±58.2	259.1±58.5	284.8±64.7	<0.001^a^
D-CAT3 score	141.2±42.5	161.3±33.4	174.6±37.1	0.001^a^

Values are mean±SD, geometric mean (25th–75th percentiles). Statistically significant. a: the one-way analysis of variance, b: Kruskal-Wallis test, c: Peason chi-square tests. Abbreviations: SBP; Systolic blood pressure, LDL; Low-density lipoprotein, mtDNA-CN; mitochondrial DNA copy number, SMMSE; Short version of Mini-Mental State Examination, LMT; Logical Memory Test, D-CAT; Digit Cancellation Test.

**Table 3:** Characteristics of study participants in stratified mtDNA-CN in women

	Women			
	mtDNA-CN			
	Low (n=37)	Middle (n=74)	High (n=37)	*P*
Age (years)	64.8±9.3	61.7±10.2	61.6±10.4	0.251^a^
SBP (mmHg)	129.2±19.6	127.2±20.6	118.8±18.5	0.054^a^
Platelet count (×10^4^/μL)	20.7±4.1	22.3±5.1	22.5±4.6	0.195^a^
LDL cholesterol (mg/dL)	122.8±32.8	122.4±28.4	132.4±33.8	0.219^a^
Smoking habit, n (%)				
never	30 (81.1)	56 (75.7)	28 (75.7)	0.506^c^
ever	6 (16.2)	12 (16.2)	4 (10.8)	
current	1 (2.7)	6 (8.1)	5 (13.5)	
Education history, n (%)				
9 years >	10 (27.8)	14 (19.5)	9 (25.0)	0.520^c^
9–12 years	12 (33.3)	33 (45.8)	18 (50.0)	
12 year <	14 (38.9)	25 (34.7)	9 (25.0)	
Relative level of mtDNA-CN	0.52±0.20	0.76±0.09	1.00±0.10	<0.001^a^
SMMSE score	28.6 (27.0–30.0)	28.5 (27.0–30.0)	29.2 (29.0–30.0)	0.066^b^
LMT score	14.1±4.7	14.9±4.9	15.6±5.7	0.427^a^
D-CAT1 score	273.6±64.8	296.0±75.4	305.2±68.4	0.141^a^
D-CAT3 score	170.6±48.6	181.9±41.8	190.6±38.2	0.135^a^

Values are mean±SD, geometric mean (25th–75th percentiles). Statistically significant. a: the one-way analysis of variance, b: Kruskal-Wallis test, c: Peason chi-square tests. Abbreviations: SBP; Systolic blood pressure, LDL; Low-density lipoprotein, mtDNA-CN; mitochondrial DNA copy number, SMMSE; Short version of Mini-Mental State Examination, LMT; Logical Memory Test, D-CAT; Digit Cancellation Test.

**Table 4:** The odds ratio and 95% confidence intervals for MCI by stratified level of mtDNA-CN in men

Cognitive function assesement	Stratified mtDNA-CN	Case, n (%)	Model 1			Model 2			Model 3	
			ORs (95% CI)	*P*		ORs (95% CI)	*P*		ORs (95% CI)	*P*
SMMSE <28										
	Low	13 (39.4)	1.73 (0.61–4.89)	0.298		0.92 (0.28–3.01)	0.894		0.90 (0.27–3.03)	0.866
	Middle	26 (38.2)	1.65 (0.67–4.10)	0.280		1.01 (0.35–2.92)	0.981		1.06 (0.35–3.21)	0.925
	High	9 (27.3)	1.00	—		1.00	—		1.00	—
	*P* for trend			0.306			0.890			0.855
LMT <25%										
	Low	14 (42.4)	2.30 (0.80–6.60)	0.121		2.18 (0.73–6.51)	0.163		2.37 (0.76–7.35)	0.136
	Middle	10 (14.7)	0.54 (0.19–1.53)	0.244		0.54 (0.18–1.61)	0.268		0.66 (0.21–2.09)	0.479
	High	8 (24.2)	1.00	—		1.00	—		1.00	—
	*P* for trend			0.087			0.104			0.090
D-CAT1 <25%										
	Low	13 (39.4)	2.93 (0.95–9.03)	0.062		2.07 (0.61–7.05)	0.243		2.24 (0.64–7.91)	0.210
	Middle	14 (20.6)	1.17 (0.40–3.37)	0.776		0.64 (0.19–2.18)	0.478		0.63 (0.18–2.28)	0.486
	High	6 (18.2)	1.00	—		1.00	—		1.00	—
	*P* for trend			0.049			0.155			0.127
D-CAT3 <25%										
	Low	13 (39.4)	3.64 (1.12–11.85)	0.032		2.88 (0.83–9.97)	0.095		3.77 (1.01–14.07)	0.048
	Middle	15 (22.1)	1.58 (0.52–4.81)	0.417		1.17 (0.35–3.90)	0.804		1.66 (0.46–6.04)	0.440
	High	5 (15.2)	1.00	—		1.00	—		1.00	—
	*P* for trend			0.025			0.067			0.038

Model 1: No adjusted. Model 2: adjusted for age and education history. Model 3: adjusted for age, education history, smoking habit, systolic blood pressure, LDL cholesterol and platelet counts.

**Table 5:** The odds ratio and 95% confidence intervals for MCI by stratified level of mtDNA-CN in women

Cognitive function assesement	Stratified mtDNA-CN	Case, n (%)	Model 1			Model 2			Model 3	
			ORs (95% CI)	*P*		ORs (95% CI)	*P*		ORs (95% CI)	*P*
SMMSE <28										
	Low	11 (29.7)	3.49 (1.00–12.24)	0.051		5.46 (1.33–22.43)	0.019		5.46 (1.28–23.38)	0.022
	Middle	20 (27.0)	3.06 (0.96–9.72)	0.059		4.90 (1.32–18.20)	0.018		4.91 (1.29–18.72)	0.020
	High	4 (10.8)	1.00	—		1.00	—		1.00	—
	*P* for trend			0.058			0.021			0.027
LMT <25%										
	Low	10 (27.0)	1.00 (0.36–2.79)	1.000		0.93 (0.32–2.71)	0.896		1.06 (0.34–3.28)	0.921
	Middle	17 (23.0)	0.81 (0.33–1.99)	0.639		0.76 (0.30–1.95)	0.574		0.83 (0.31–2.23)	0.704
	High	10 (27.0)	1.00	—		1.00	—		1.00	—
	*P* for trend			1.000			0.896			0.911
D-CAT1 <25%										
	Low	13 (35.1)	2.32 (0.80–6.73)	0.121		1.73 (0.52–5.73)	0.372		1.86 (0.49–7.13)	0.365
	Middle	17 (23.0)	1.28 (0.48–3.42)	0.625		1.26 (0.42–3.80)	0.683		1.29 (0.37–4.45)	0.688
	High	7 (18.9)	1.00	—		1.00	—		1.00	—
	*P* for trend			0.110			0.363			0.348
D-CAT3 <25%										
	Low	15 (40.5)	2.92 (1.02–8.37)	0.046		2.92 (0.89–9.62)	0.078		2.64 (0.70–10.00)	0.152
	Middle	15 (20.3)	1.09 (0.40–2.96)	0.866		1.45 (0.47–4.42)	0.517		1.15 (0.32–4.10)	0.827
	High	7 (18.9)	1.00	—		1.00	—		1.00	—
	*P* for trend			0.034			0.066			0.114

Model 1: No adjusted. Model 2: adjusted for age and education history. Model 3: adjusted for age, education history, smoking habit, systolic blood pressure, LDL cholesterol and platelet counts.
